# Optimizing Asphalt Surface Course Compaction: Insights from Aggregate Triaxial Acceleration Responses

**DOI:** 10.3390/ma16227239

**Published:** 2023-11-20

**Authors:** Zhi Zhang, Hancheng Dan, Songlin Li, Wenfeng Li

**Affiliations:** School of Civil Engineering, Central South University, Changsha 410075, China; zhizhang@csu.edu.cn (Z.Z.); 234811173@csu.edu.cn (S.L.); wenfengli@csu.edu.cn (W.L.)

**Keywords:** asphalt surface course, vibration compaction, aggregate acceleration, compaction degree, SmartRock sensor

## Abstract

The compaction quality of asphalt surface courses has a significant impact on the overall performance of asphalt pavements, but the dynamic response and compaction degree variations of different asphalt surface courses (top, middle, and bottom surface courses) during vibrational compaction have still received limited research. SmartRock sensors can be utilized to monitor aggregate acceleration in real-time. This study aims to address this gap using SmartRock sensor technology to further understand the compaction mechanisms of different asphalt surface courses from a particle perspective, as well as the relationship between aggregate acceleration and compaction degree. The results indicate that the rolling of steel drums induces a significant alteration of the aggregate acceleration along the roller’s rolling direction, primarily resulting in horizontal shearing in that direction. As distance increases, vibration waves gradually attenuate on both sides of vibrating drums, and surface course thickness and gradation significantly affect acceleration amplitude. There is a linear correlation between triaxial aggregate acceleration and compaction degree, with the vertical correlation being the strongest. Finally, an empirical relationship between triaxial acceleration and pavement compaction degree was established, providing a basis for predicting the asphalt surface course density. These findings enhance our understanding of pavement compaction mechanisms and promote innovation in asphalt pavement compaction and quality control methods.

## 1. Introduction

The asphalt surface course is located directly above the pavement base course and plays an important role in bearing traffic loads and protecting the base structure [1]. During construction, the pavement achieves its load-bearing function through various types of external compaction forces, thereby impacting its performance [2]. The performance of the pavement, including durability, crack resistance, and skid resistance, directly impacts its service life and driving safety. Adequate compaction of the asphalt surface course can effectively reduce the stress on the pavement base, providing a solid foundation for the service performance of the asphalt pavement. Insufficient compaction of the asphalt pavement can cause damage to the entire pavement structure, affecting the safety and service life of the road [3], and increase traffic noise emissions [4]. Therefore, the degree of compaction of the asphalt pavement is crucial when it comes to preserving the stability and endurance of the complete pavement structure.

The compaction of asphalt pavement involves the use of vibration or compaction action to tightly compact materials, thereby enhancing their density and strength [5,6]. In the construction of asphalt pavement, pneumatic tire rollers, vibratory rollers, and static steel rollers are indispensable equipment. In traditional pavement compaction methods, roller operators often cannot accurately perceive the state of the compacted material and obtain timely feedback. This means that they could only rely on the knowledge and experience of engineers to ensure uniform and effective compaction [7]. Most existing methods for ensuring the quality of compaction, such as non-nuclear density gauges, coring, ground penetrating radar, etc., can only evaluate the compaction quality through post-detection, and cannot promptly detect and adjust problems during construction [8]. Therefore, these methods have certain limitations for efficient and real-time quality control.

The technology known as intelligent compaction (IC) is emerging and provides accurate and timely compaction quality control. IC utilizes advanced modern rollers that come with built-in measurement systems, global positioning systems, infrared temperature sensors, and onboard reporting systems [9]. With its advantages of real-time monitoring, precise positioning, and automated adjustment, this technology can promptly identify and correct issues such as insufficient or excessive compaction and low pavement uniformity, while providing real-time quality control guidance, thereby improving the quality and performance of the pavement. However, IC technology still has some limitations. Firstly, the current measured compaction control value (CCV) represents the combined stiffness of several layers beneath the roller and cannot measure the stiffness of individual layers [10,11,12]. Secondly, due to the complex interaction between vehicle surfaces and the influence of asphalt binder viscosity on the rebound behavior of the drum, there is inconsistent correlation between IC measurement values and density [11]. In conclusion, the application of IC technology requires a necessary understanding and knowledge of the compaction mechanism of asphalt pavement materials.

Superpave gyratory compaction (SGC) is a widely used method for simulating on-site compression in the laboratory, applying both shear and compressive forces to asphalt mixture [5,13,14]. Tashman et al. [13] utilized X-ray computed tomography technology and found that certain areas of SGC specimens exhibited similar void distribution to field cores. However, previous studies [15,16,17] have indicated that there are significant differences in the mechanical characteristics between SGC specimens and field cores, possibly due to varied compaction mechanisms and particle interactions. In recent years, some researchers have employed SmartRock sensors in SGC tests of asphalt mixtures [6,18,19,20]. It is worth mentioning that the SmartRock sensor is a commercial product. Wang et al. [6,19] analyzed particle motion characteristics (rotation and acceleration) during SGC compaction using SmartRock and found a close correlation between particle rotation and mixture density changes, enabling monitoring of the compaction status of the mixture. Dan et al. [18] utilized SmartRock to study the compaction characteristics of asphalt mixtures and identified an inherent connection between particle responses and compaction properties. These studies demonstrate that SmartRock can investigate the compaction mechanism of asphalt mixtures from the perspective of particle motion. Additionally, SmartRock has been applied in field compaction studies of asphalt pavements [21,22,23]. Dan et al. [21] utilized SmartRock to investigate the dynamic response of asphalt pavements during the compaction progress and found a close connection between the dynamic responses of asphalt mixture and the degree of compaction. Wang et al. [22] used SmartRock to monitor particle response to different types of compaction equipment during compaction and found that rubber wheels could better simulate kneading effects. While the aforementioned studies verify the effectiveness of SmartRock in monitoring particle motion responses during asphalt pavement compaction, the research on compaction of asphalt pavement using SmartRock sensors is still in its early stages. Further research and calibration using SmartRock sensors are needed to investigate the relationship between aggregate acceleration responses and compaction characteristics of different asphalt surface courses during the on-site compaction.

In summary, traditional methods of asphalt pavement compaction suffer from the problem of operators having difficulty accurately perceiving the compacted material state and obtaining timely feedback. Furthermore, significant breakthroughs have been made in the field of intelligent compaction technology for automatic quality control; however, there are still problems with the inability to measure the stiffness of individual asphalt pavement layers and inconsistency between IC measurement values and density. Although SmartRock has been successfully applied in SGC tests for asphalt mixtures and field compaction of asphalt pavement, the study of asphalt pavement compaction utilizing SmartRock sensors is still in its early stages. Further research and calibration are needed to investigate the relationship between aggregate acceleration responses and compaction characteristics of different asphalt surface courses during the on-site compaction using SmartRock sensors. This study aims to understand the compaction mechanism of the asphalt pavement from a particle perspective by monitoring the aggregate acceleration response and compaction degree variations in the top, middle, and bottom surface courses of asphalt pavement. In order to further investigate the aggregate acceleration characteristics and compaction characteristics of asphalt pavement, as well as the correlation between the two, it will contribute to deepening a scientific basis for compaction quality prediction, and thus promote the development of IC.

## 2. SmartRock Sensor

### 2.1. Composition, Performance, and Data Collection

**Figure 1:**
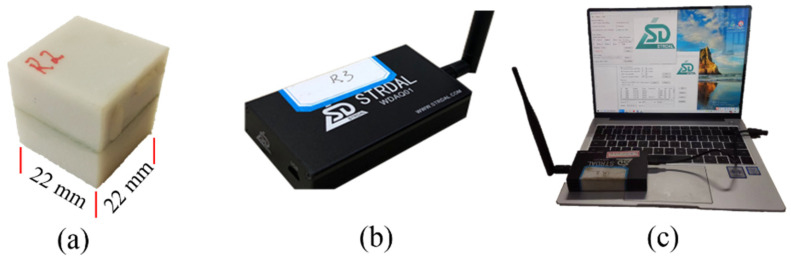
Data acquisition System: (**a**) SmartRock, (**b**) signal receiver, and (**c**) signal processing software.

SmartRock is an ultra-small and high-temperature resistant sensor, with dimensions similar to the aggregate in asphalt mixtures, as shown in Figure 1a. Due to its successful application in laboratory and field compaction studies of asphalt mixtures, this study utilizes SmartRock to monitor the particle movement characteristics during pavement compaction, aiming to facilitate the understanding of compaction mechanisms of asphalt mixtures. The outer shell of a SmartRock exhibits excellent adhesion properties, ensuring reliable performance in high-temperature compaction environments by tightly bonding with the asphalt material. Internally, SmartRock is equipped with a triaxial accelerometer, gyroscope, triaxial magnetometer, and temperature sensor, enabling real-time, high-precision recording of parameters such as standard time, acceleration, quaternion in Earth coordinates, triaxial contact stress, and internal temperature. The data acquisition system consists of three modules: SmartRock, signal receiver, and signal processing software, as shown in Figure 1. During compaction, SmartRock collects data and transmits it to the portable signal receiver, as depicted in Figure 1b, via low-energy Bluetooth. Subsequently, the data is uploaded in real-time to the signal processing software on a computer (Figure 1c) for storage and utilization. Furthermore, the small size of SmartRock and its Bluetooth transmission technology can reduce interference during compaction.

### 2.2. Coordinate System Transformation

**Figure 2:**
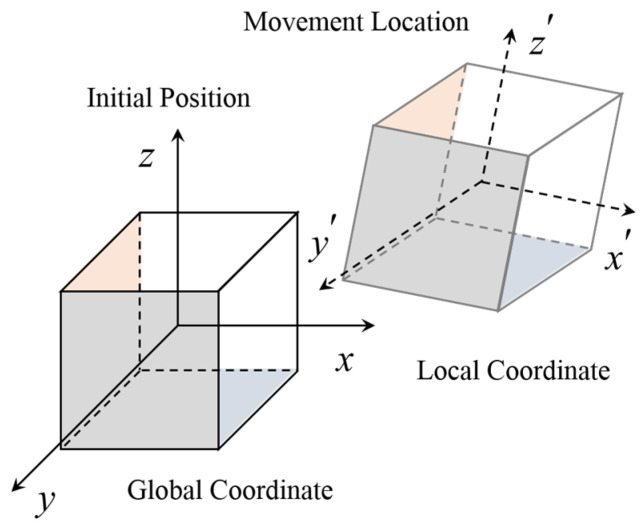
The coordinate system of SmartRock.

The motion data of particles is recorded when SmartRock is applied, and this data is based on the local coordinate system (*x’*, *y’*, and *z’*) connected to its own rigidity. Subsequently, the data is transformed into the global coordinates (*x*, *y*, and *z*) for further comparison and analysis. To achieve this transformation, SmartRock needs to consider the conversion relationship between the local and global coordinate systems (Figure 2).

Quaternions are mathematical constructs used to represent the orientation of a rigid body in three-dimensional space. It is composed of one real part and three imaginary parts, often represented as $q=q_{0}+q_{1}i+q_{2}j+q_{3}k$, where $q_{0}$, $q_{1}$, $q_{2}$, and $q_{3}$ are real numbers and *i*, *j*, and *k* are imaginary units. The SmartRock employs a quaternion-based rotation matrix $R_{q}(q)$ to encode the posture of a rigid body. The rotation matrix is represented as shown in Equation (1).

(1) $$\left[\begin{array}{ccc} q_{0}^{2}+q_{1}^{2}-q_{2}^{2}-q_{3}^{2} & 2q_{1}q_{2}+2q_{0}q_{3} & 2q_{1}q_{3}-2q_{0}q_{2}\\ 2q_{1}q_{2}2q_{0}q_{3} & q_{0}^{2}-q_{1}^{2}+q_{2}^{2}-q_{3}^{2} & 2q_{2}q_{3}+2q_{0}q_{1}\\ 2q_{1}q_{3}+2q_{0}q_{2} & 2q_{2}q_{3}-2q_{0}q_{1} & q_{0}^{2}-q_{1}^{2}-q_{2}^{2}+q_{3}^{2} \end{array}\right]$$

where $q_{0}$, $q_{1}$, $q_{2}$, and $q_{3}$ represent the coefficients of the unit quaternions collected by SmartRock.

SmartRock can transform data in the local coordinate system to the global coordinate system using the rotation matrix $R_{q}{\left(q\right)}^{T}$. In other words, multiplying the rotation matrix with the vector represented in the local coordinate system yields the same vector represented in the global coordinate system. The formula for coordinate vector transformation is as follows:

(2) $$z=R_{q}{\left(q\right)}^{T}z^{\prime }$$

where *z* ϵ *R*^3^ denotes a vector within the global coordinate system, while *z′* ϵ *R*^3^ denotes a vector in the local coordinate system.

SmartRock collects quaternion and acceleration data based on the local coordinate system in the process of compaction. Then, according to Equations (1) and (2), the collected local coordinate system acceleration is converted into global coordinate system acceleration for further analysis. In other words, all the data presented in this study rely on the use of the global coordinate system.

## 3. Materials and Experiments

### 3.1. Materials

**Figure 3:**
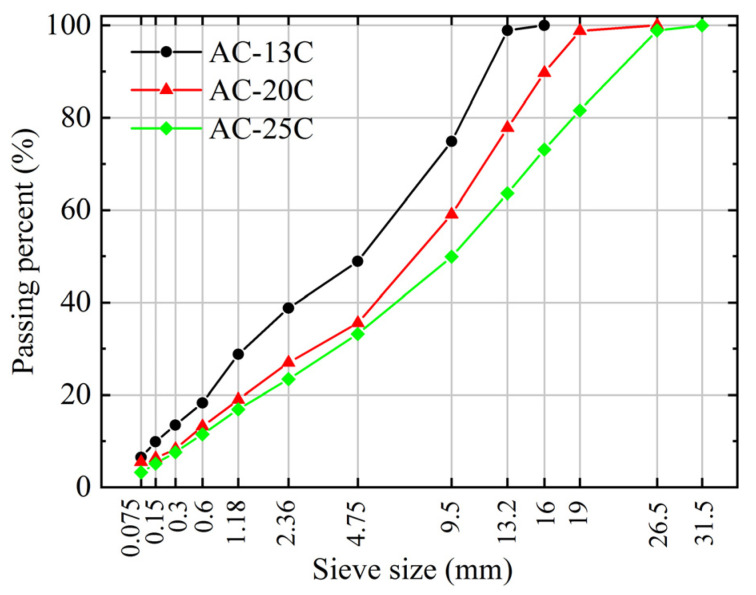
The gradation curves of three asphalt mixtures.

A field investigation was carried out in the process of constructing a new highway in Guizhou Province, China, to monitor the characteristics of aggregate movement and pavement compaction. Asphalt pavement typically consists of multiple layers, including the asphalt surface course, base course, subbase course, and road base. The three constituent structures of the asphalt surface course or wearing course, namely the top surface course, middle surface course, and bottom surface course. The study focused on asphalt surface courses with different thicknesses in the Zheng’an to Xishui section of the project, including the top, middle, and bottom surface courses with design thicknesses of 4 cm, 6 cm, and 8 cm, respectively. Three types of the asphalt mixture, AC-13C, AC-20C, and AC-25C, designed according to the Marshall design method, were selected for the construction of the top, middle, and bottom surface courses of the asphalt pavement, respectively. The letter “C” in AC-13C stands for coarse-graded asphalt mixture. The classification of the coarse-graded asphalt mixture and fine-graded asphalt mixture is determined by the passing rate of the critical sieve size. For the AC-13C, AC-20C, and AC-25C, the critical sieve sizes are 2.36 mm, 4.75 mm, and 4.75 mm, respectively, with passing percentages of less than 40%, less than 45%, and less than 40%, respectively. Coarse aggregates (particle size larger than 4.75 mm) used in AC-13C and AC-20C mixtures were made of basalt crushed stone, while limestone crushed stone was used in the AC-25C mixture. Fine aggregates (particle size smaller than 4.75 mm) for all mixtures were made of limestone crushed stone. The specific aggregate gradations can be seen in Figure 3. SBS (I-D) modified asphalt was used in both AC-13C and AC-20C asphalt mixtures, while 70# A-grade road petroleum asphalt was used in AC-25C asphalt mixture. Additionally, limestone mineral powder was used in all asphalt mixtures. The optimal asphalt content, voids filled with mineral aggregates (VMA), and voids filled with asphalt (VFA) of three asphalt mixtures are shown in Table 1.

### 3.2. Field Experiment

**Figure 4:**
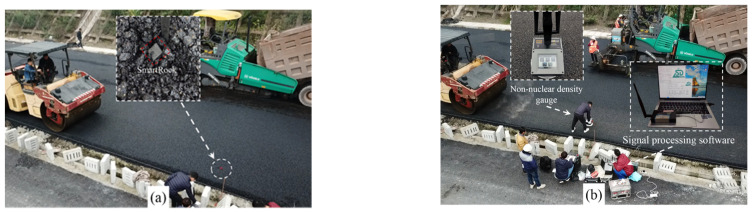
Compaction experimental site: (**a**) embedding SmartRock in asphalt pavement and (**b**) data monitoring.

The field experiment for asphalt pavement compaction is illustrated in Figure 4. After the paver completes the paving of the asphalt mixture, the test personnel will embed the SmartRock into the asphalt mixture and mark its location, as shown in Figure 4a. Furthermore, when embedding the SmartRock into the asphalt surface course, its local coordinate system *x’*, *y’*, and *z’* will correspond to the road cross-section, vehicle driving direction, and the direction perpendicular to the road surface, respectively, to facilitate subsequent data processing. The field experiment utilized the Dynapac CC624HF double steel drum roller to compact the asphalt mixture after paving. The vibration drum operated in a low frequency (51 Hz) and high amplitude (0.8 mm) mode, with a weight of 6000 kg and an excitation force of 166 kN. In order to retain the information in the original signal completely, SmartRock’s sampling frequency was established at 104 Hz. The monitoring of the pavement rolling process is depicted in Figure 4b, where the SmartRock sensor recorded the motion data of particles during compaction and transmitted them in real-time to a computer-based signal processing software via a portable signal receiver for storage and analysis. Additionally, after each pass of compaction by the roller, the compaction degree of the asphalt pavement was assessed by employing a non-nuclear density gauge, serving as one of the indicators to evaluate compaction quality.

### 3.3. Experiment Design

**Figure 5:**
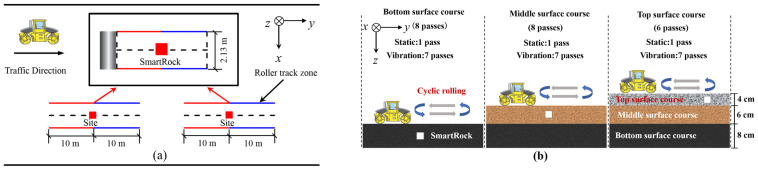
Test scheme: (**a**) SmartRock layout and (**b**) compaction program.

In this project, SmartRock and compactness monitoring were conducted on the top, middle, and bottom surface courses of asphalt pavement, respectively. The on-site experiment design is illustrated in Figure 5. To ensure effective monitoring of the dynamic response of the aggregate in the asphalt surface courses, SmartRock was embedded near the middle-bottom portion of the middle and bottom surface courses. Additionally, considering the thinness of the top surface course, SmartRock was embedded near the bottom of the top surface course to prevent impact from the roller and ensure its normal operation under vibratory compaction. The layout of the sensors during on-site pavement compaction is shown in Figure 5a, with SmartRock initiating data collection 10 m away from the roller and continuing until the repeated compaction of that section is finished. Furthermore, three sites were selected for SmartRock and compaction testing in each surface course, with adequate distance between them to avoid mutual interference.

The compaction of the asphalt surface course was carried out in the order of the bottom, middle, and top surface courses, as shown in Figure 5b. During on-site operation, the front drum of the double steel drum roller performed vibration compaction, while the rear drum performed static compaction. The compaction process for the middle, bottom surface courses is the same, with a total of eight passes. This means that the roller first applies static compaction to the paved asphalt surface course in a forward driving mode, and then alternates between backward and forward driving modes to perform seven passes of vibratory compaction on the SmartRock embedment site. Additionally, due to the thinner thickness of the top surface course, its rolling process differs from the middle, bottom surface courses, with a total of six passes. Initially, the roller performed one pass of static compaction, followed by five passes of vibratory compaction using alternating forward and backward driving modes on the SmartRock embedment site.

## 4. Results and Discussions

### 4.1. Original Triaxial Acceleration Responses

**Figure 6:**
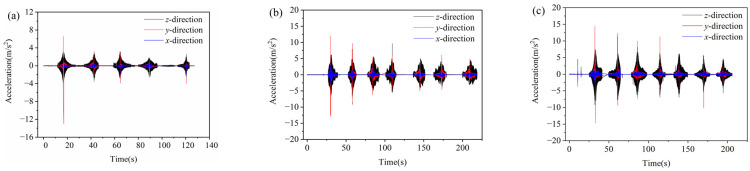
Triaxial acceleration responses of aggregates during compaction: (**a**) top surface course, (**b**) middle surface course, and (**c**) bottom surface course.

During compaction of asphalt pavement, the SmartRock sensor interacts with the surrounding aggregates to form a force-bearing framework. Its motion mode (rotation and translation) is most similar to that of the surrounding aggregate, which can describe the movement of the aggregate during compaction. The three-axis (*x*, *y*, and *z*) acceleration responses of SmartRock in the top, middle, and bottom surface courses of the asphalt pavement during compaction are shown in Figure 6. It can be observed that the aggregates continuously vibrate in three axes during compaction, and their acceleration responses have similar motion patterns in the three surface courses, with the *z*-axis having the maximum acceleration while the *x*-axis has the minimum. This is because the asphalt mixture undergoes continuous compaction due to vibration force, and the vertical movement of the aggregates dominates, and the aggregates are subjected to the mutual squeezing of the surrounding particles, resulting in horizontal movement (horizontal shear). Among them, the aggregates are subjected to different external constraints in the axial (*x*-axis) and radial (*y*-axis) directions of the vibration drum, with greater constraint in the *x*-axis direction than in the *y*-axis direction, leading to a larger acceleration response on the *y*-axis than on the *x*-axis. Therefore, the horizontal shear effect of the mixture mainly occurs in the rolling direction (*y*-axis). Based on the above analysis, it can be concluded that vertical compression and horizontal shear are the main causes of asphalt mixture densification, with vertical compression playing the dominant role. Additionally, when the vibration drum comes into contact with the SmartRock sensor, the *y*-axis acceleration undergoes a significant sudden change. It should be noted that there is no significant change in the acceleration on the *z*-axis and *x*-axis. This indicates that the impact force of the rolling wheel (*y*-axis) causes a sudden increase in acceleration. Therefore, the rolling drum has a noticeable interfering effect on the acceleration in the rolling direction of rollers, making the horizontal shear in the rolling direction more significant.

### 4.2. Filtering of Raw Acceleration

**Figure 7:**
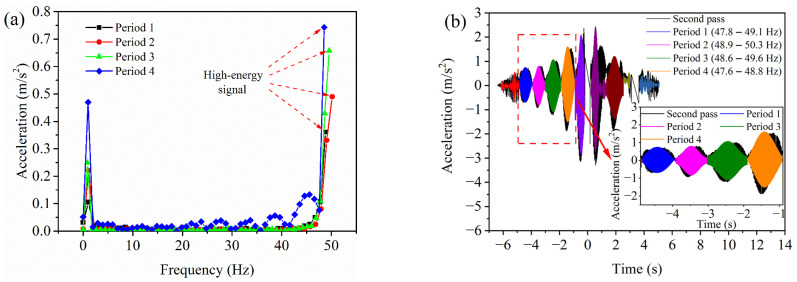
The FFT method: (**a**) spectrogram and (**b**) time-domain post-processing data.

The vibrations generated by on-site machinery such as pavers, vibratory rollers, and pneumatic-tired rollers produce noise signals that affect the accuracy and stability of acceleration data. To address this issue, the raw acceleration can be filtered using fast Fourier transform (FFT) to extract high-energy signal data induced by the vibrating drum. Taking the filtering process of the second pass acceleration of the vibratory roller as an example, the time-domain raw acceleration of four typical time periods is transformed into frequency-domain raw acceleration (i.e., spectrogram) using FFT, as shown in Figure 7a. It is noteworthy that a logarithmic representation of the amplitude is adopted on the vertical axis in Figure 7a to better observe periodic signals in low-amplitude noise, and its horizontal axis is shown on a normal numerical scale. The time-domain raw acceleration is usually a multi-frequency signal composed of high-energy signals of different frequencies and low-energy noise. Each frequency in the frequency-domain original acceleration corresponds to an acceleration amplitude. Therefore, through spectral analysis, the frequency corresponding to the maximum acceleration amplitude signal in the spectrogram (i.e., high-energy signal frequency) can be identified. According to Figure 7a, in the frequency domain, the raw acceleration of a vibrating roller typically exhibits a high-energy signal frequency that is close to the fundamental frequency of the roller’s vibration (51 Hz). Additionally, for the time-domain raw acceleration of each time period, a band-pass filter with an appropriate frequency range (within 2 Hz) centered around the high-energy signal frequency is employed for filtering, thereby obtaining post-processed time-domain data, as shown in Figure 7b. The horizontal and vertical axes in Figure 7b are shown as normal numerical scales. In summary, by using the FFT to convert the time-domain original data into the frequency-domain original data, the high-energy signal frequency is obtained. Based on this frequency, the time-domain original acceleration is processed by band-pass filtering, thus capturing the acceleration signals induced by the vibrating roller.

### 4.3. Variation of Compaction Degree

**Figure 8:**
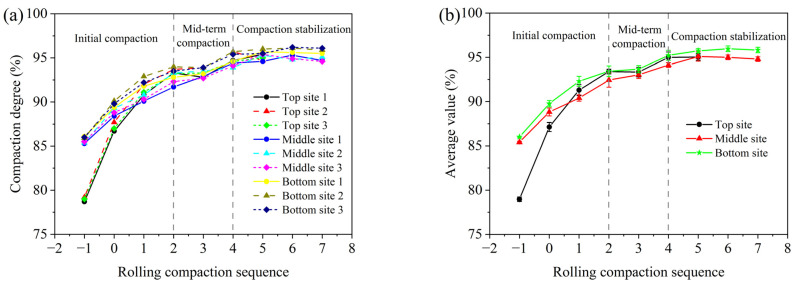
The compaction degree of three asphalt surface courses with different rolling passes: (**a**) original value and (**b**) average value and variance.

Figure 8 depicts the compaction degree curves of three asphalt surface courses under different numbers of rolling passes, aiming to investigate the variation pattern of their compaction degree with rolling passes. Figure 8a,b present the compaction degrees of nine testing sites, as well as the average value and variance of the compaction degrees for each layer (three test sites). The sequence numbers −1 and 0 represent the compaction degrees after paving with the mixture and after initial static compaction, respectively, while other sequence numbers indicate the times of vibrating rolling passes. Based on the analysis of results, the compaction process can be divided into three phases: initial compaction, mid-term compaction, and compaction stabilization. In the initial compaction stage, the compaction degree increases significantly due to the loose state and high-temperature plasticity of the mixture (increase by 14.6% for the top surface course, 7.15% for the middle surface course, and 7.45% for the bottom surface course). In the mid-term compaction stage, the aggregates are continuously rearranged under the action of vibration compaction force, and the aggregate framework can partially bear the compaction force and gradually increase the compaction density. However, as the temperature decrease, the material’s mechanical performance is between high-temperature plasticity and viscoelasticity, leading to a weakened compactability. Therefore, the increment (1.65% for the top surface course, 1.75% for the middle surface course, and 1.7% for the bottom surface course) of the compaction degree slows down. In the compaction stabilization stage, a stable skeleton structure is formed within the asphalt mixture, exhibiting viscoelasticity and being able to bear most of the external pressure, making it difficult to further compact the material. Therefore, the externally applied compaction force cannot effectively increase the density of the mixture. Furthermore, the number of roller passes at the stable compaction degree should be considered as the optimal number of passes, representing the fewest passes to achieve the best compaction state. As shown in Figure 8b, each surface course reaches a stable compaction platform at different critical numbers of roller passes, with the top surface course at the fourth pass, the middle surface course at the fifth pass, and the bottom surface course at the sixth pass. It is worth mentioning that odd numbers of rolling passes are typically not arranged in roadwork, as this can result in uneven compaction of asphalt mixtures. To ensure a more uniform and stable road surface, it is common practice to schedule an even number of roller passes during road construction. Consequently, the optimal number of vibrating compaction passes for the top, middle, and bottom surface courses are the fourth, sixth, and sixth passes, respectively. It is worth noting that the optimal number of vibrating compaction passes applies only to the specific roller equipment, compaction method, and asphalt mixture performance mentioned in this paper.

### 4.4. Rolling Speed of the Roller

**Figure 9:**
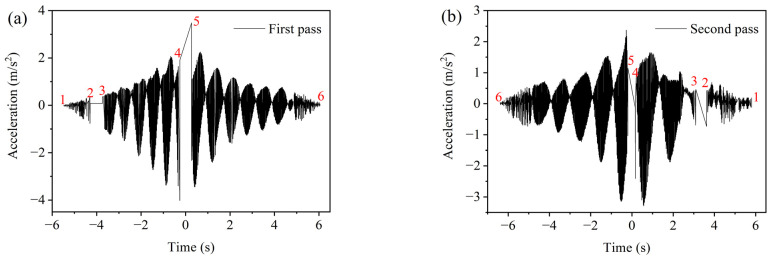
Raw acceleration of *z*-axis in two driving modes: (**a**) backward driving and (**b**) forward driving.

During the on-site construction of asphalt pavements, the roller operates in two modes—forward driving and backward driving—to compact the selected road area. In order to monitor the actual rolling speed of the roller and calculate the distance between the vibration drum and SmartRock based on time, the time curve of the *z*-axis raw acceleration for the vibratory roller is shown in Figure 9 under both driving modes. It is worth mentioning that the red numbers (1 to 6) in Figure 9a,b represent time division markers, facilitating the determination of the time each wheel of the roller passes the SmartRock measurement sites. Due to the obstruction of the steel drum on top of the SmartRock sensors, the Bluetooth signal of the sensor in the asphalt mixture cannot be obtained. However, SmartRock can monitor the time it takes for the static drum (*T*_23_ and *T*_32_) and the vibrating drum (*T*_45_ and *T*_54_) to pass over it, as well as the time between the two drums (*T*_34_ and *T*_43_), to determine the speed of the roller. Therefore, the rolling speed of the roller can be calculated based on the acceleration time curve and the wheelbase of the roller using Equations (3) and (4).

*V_backward_* = *S*/(0.5 × (*T*_23_ + *T*_45_) + *T*_34_) (3)

*V_forward_* = *S*/(0.5 × (*T*_32_ + *T*_54_) + *T*_43_) (4)

where *V_backward_* and *V_forward_* represent the respective speeds at which the roller moves backward and forward, respectively (m/s), while *S* (3690 mm) denotes the wheelbase of the Dynapac roller.

**Figure 10:**
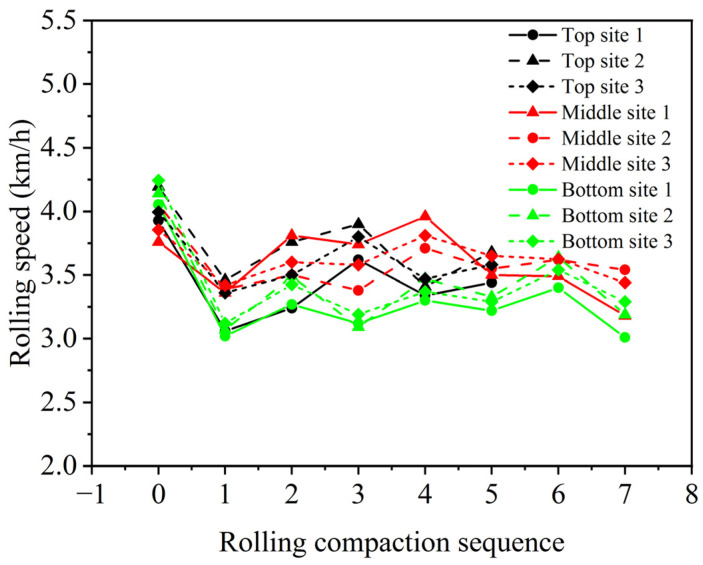
Rolling speeds of the roller.

The rolling speeds of the roller with different numbers of roller passes for three asphalt surface courses are shown in Figure 10. The rolling speed of the roller ranges from 3.01 km/h to 4.24 km/h, and the speed of the static drum is significantly higher than that of the vibrating drum. Therefore, SmartRock is capable of monitoring the rolling speed of the roller for subsequent calculations of the distance between the vibrating drum and SmartRock.

### 4.5. Acceleration of Aggregates on Both Sides of Vibrating Drum

**Figure 11:**
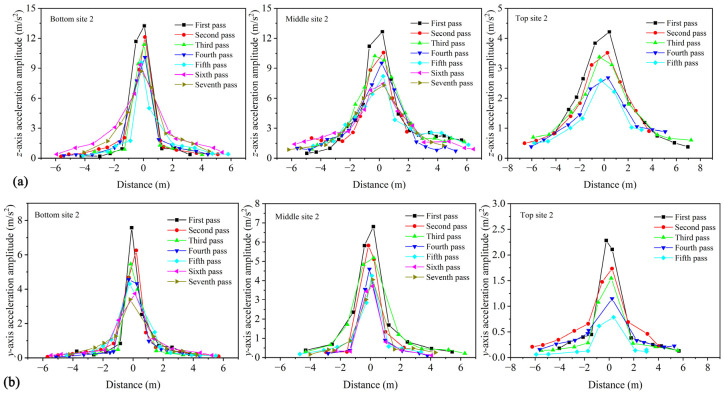

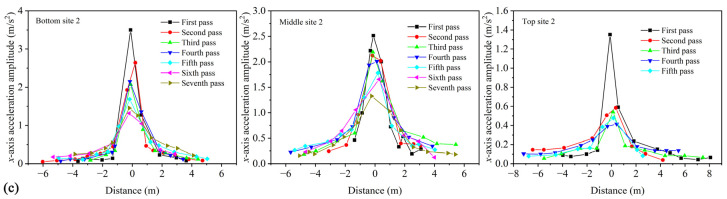
Acceleration amplitude with different rolling passes varies with distance: (**a**) *z*-axis, (**b**) *y*-axis, and (**c**) *x*-axis.

When the vibrating roller applies compaction force, it generates a vibration wave on the asphalt surface course, which propagates within the asphalt surface course. By measuring the acceleration of the aggregates, the propagation characteristics of the vibration wave in the asphalt surface course can be understood. The acceleration responses during compaction is filtered using FFT to obtain the variation of three-axis accelerations of aggregates on both sides of the vibratory drum with distance under different number of rolling passes (Figure 11). The horizontal axis in the figure represents distance, which is calculated based on time and the corresponding velocity for each pass. The positive and negative axes indicate the direction of the vibratory roller approaching and leaving the SmartRock, respectively. It should be noted that, for simplicity, only one measuring site for acceleration analysis was selected for each surface course in the diagram. From Figure 11, it can be observed that the variation of three-axis acceleration of aggregates on both sides of the vibratory drum with distance follows a normal distribution trend under different number of rolling passes, indicating that the vibration wave gradually attenuates with the increasing propagation distance on both sides of the vibratory drum. The maximum acceleration occurs at the bottom of the vibratory drum, while the acceleration on both sides significantly attenuates. This indicates that the compaction effect of the vibratory roller mainly focuses on the compacting area of the drum, and the aggregate particles on both sides are not significantly affected by loosening. This is mainly because the vibration wave in the aggregate is dampened by factors such as inter-particle movement and lubricating effects of the high-temperature asphalt binder during propagation, resulting in gradual attenuation of the vibration wave with increasing distance. By comparing the magnitudes of three-axis acceleration on both sides of the vibratory drum for the three surface courses, it can be concluded that the bottom surface course has the highest acceleration, followed by the middle surface course, and the top surface course has the lowest acceleration. During compaction, the aggregates move and rotate with the assistance of lubricating asphalt binder, interacting with each other to maintain structural stability. Under the same compaction energy, the acceleration of aggregates decreases with the reduction in surface course thickness and gradation. This indicates that surface course thickness and material gradation have a significant influence on the vibratory acceleration of SmartRock.

### 4.6. Acceleration of Aggregates at the Bottom of Vibrating Drum

Based on Figure 9, it can be observed that the SmartRock embedded in the asphalt surface course cannot accurately capture the acceleration response of the sensor when the vibrating drum acts on its top. Additionally, as discussed in Section 4.5, the triaxial acceleration of the aggregate on both sides of the vibrating drum follows a normal distribution pattern as the distance changes. Therefore, a non-linear fitting using a Gaussian function [24] (Equation (5)) is employed to estimate the triaxial acceleration amplitude of the aggregate at the bottom of the vibrating drum, based on the curves of acceleration amplitude changing with distance for each rolling pass for the three asphalt surface courses in Figure 11.

(5) $$y=y_{0}+A\times e^{\left(-0.5\times {\left(\frac{x-x_{c}}{w}\right)}^{2}\right)}$$

where *y_0_*, *A*, *x_c_*, and *w* are regression constants.

**Figure 12:**
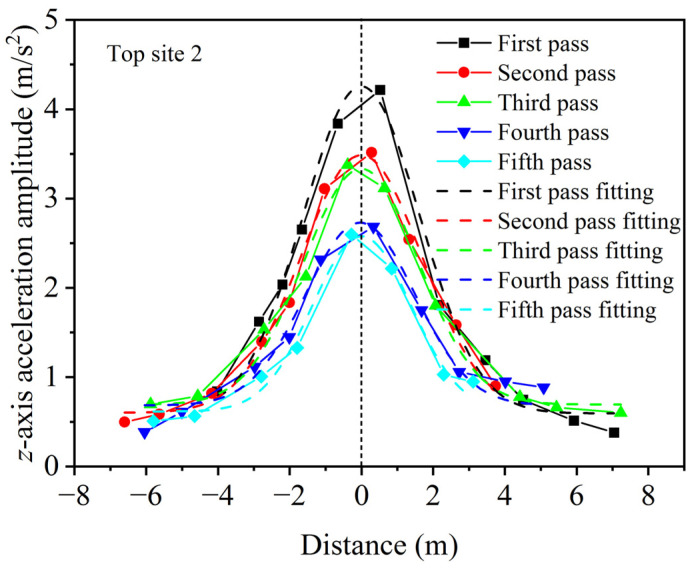
Gaussian fitting of acceleration amplitudes varying with distance.

According to the fitting based on the Gaussian function for the data in the *z*-axis of the top surface course in Figure 11, the fitting results are shown in Figure 12. By comparing the fitting curve with the experimental curve, it can be observed that the Gaussian function can effectively fit the trend of the triaxial acceleration of aggregates on both sides of the vibrating drum with respect to distance, thereby obtaining the triaxial acceleration amplitude of the aggregates at the bottom of the vibrating drum.

**Figure 13:**
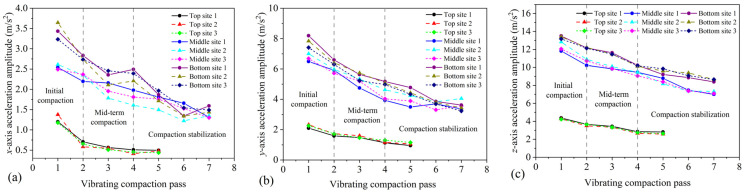
Variation of acceleration amplitudes with different vibrating compaction passes: (**a**) *x*-axis, (**b**) *y*-axis, and (**c**) *z*-axis.

Figure 13 illustrates the variation of aggregate acceleration at the bottom of the vibrating drum with the number of vibrating rolling passes. The study reveals that the aggregate acceleration gradually decreases and stabilizes with the increase in the number of vibrating rolling passes in three asphalt surface courses. This indicates that during vibration compaction, the aggregates in the asphalt mixture become interlocked through movement and rotation, ultimately forming an interlocking state of the aggregate skeleton structure. In this state, the acceleration response of the aggregates tends to stabilize. Overall, the acceleration curve can microscopically reflect the evolution of the internal skeleton structure of the asphalt pavement during compaction. Theoretically, under vibration compaction, the aggregates in the asphalt mixture continuously rotate and move, reaching a critical state of compaction known as the aggregate locking point. Therefore, the number of passes at which the aggregate acceleration stabilizes (aggregate locking point) can be considered as the optimal compaction number. Based on the data in Figure 13, the optimal number of vibrating rolling passes for the middle, bottom surface courses, as shown by the acceleration curves for the *x*- and *y*-axes, is the sixth pass, while for the *z*-axis acceleration curve, it is the seventh pass. By comparing with the optimal rolling number obtained from the compaction analysis in Section 4.3, it is found that the compaction degree reaches a stable plateau earlier than the acceleration response with the increase in the number of passes. This indicates that the acceleration indicators of the middle, bottom surface courses lag behind the compaction degree indicator. In addition, the optimal rolling passes for the *y*-axis and *z*-axis accelerations in the bottom surface course are consistent with the compaction degree, both being the fourth pass. This indicates that the acceleration indicators for the *y* and *z*-axis of the bottom surface course are consistent with the compaction degree indicators. In conclusion, the characteristics of aggregate acceleration variation can serve as control parameters for monitoring compaction and as relevant indicators for evaluating changes in the aggregate skeleton and compaction conditions.

### 4.7. Correlation between Triaxial Acceleration and Compaction Degree

**Figure 14:**
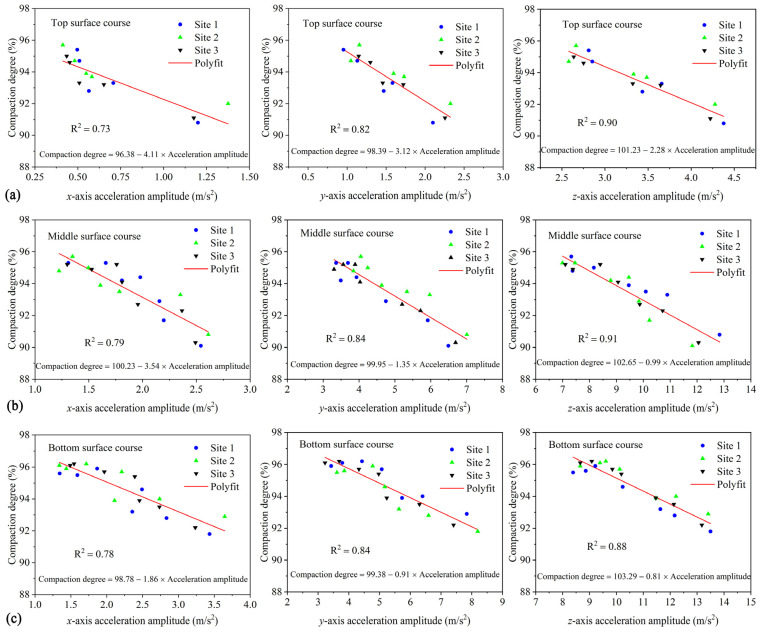
The relation between acceleration amplitude and compaction degree: (**a**) top surface course, (**b**) middle surface course, and (**c**) bottom surface course.

In order to investigate the relationship between aggregate acceleration and compaction degree of asphalt mixtures, and to use the aggregate acceleration as an indicator reflecting the compaction situation of asphalt pavement, a linear correlation analysis was performed between the triaxial acceleration of the aggregates in three asphalt surface courses and the respective compaction degree. The correlation curves and empirical relationship formulas are shown in Figure 14. These empirical formulas can provide a basis for predicting the compaction degree of the top, middle, and bottom surface courses of the same pavement structure system, which can help improve the quality control and decision-making of asphalt pavements. It is worth noting that the correlation coefficients *R^2^* range of the fitting relationship formulas in the vertical direction (*z*-axis), rolling direction (*y*-axis), and road transverse direction (*x*-axis) are 0.88~0.91, 0.82~0.84, and 0.73~0.79, respectively. It can be observed that the correlation in the vertical direction is the strongest, while the correlation in the road transverse direction is the weakest. Therefore, it is recommended to use the linear correlation formula between vertical acceleration and compaction degree for prediction in future studies. The compaction degree of the three asphalt surface courses can be calculated using the following formula: compaction degree = *a* − *b* × vertical acceleration amplitude, where *a* and *b* are fitting parameters. The specific numerical values of *a* and *b* can be found in Figure 14. In addition, by comparing the absolute values of the slope of the fitted curves for different asphalt surface courses, it is found that the absolute value of the slope is the smallest for the bottom surface course and the largest for the top surface course. This implies that during compaction, the acceleration reduction range is the largest for the bottom surface course and the smallest for the top surface course. During compaction, the skeleton in the mixture undergoes mutual extrusion and movement, and a stable skeleton structure will hinder the movement of coarse aggregates inside the mixture, resulting in a compacted and stable state. A smaller reduction range of acceleration during compaction indicates that it is easier to achieve a dense skeleton structure and compaction stability. Therefore, the slope of the fitted curve can serve as an indicator for assessing the ease of forming a compact skeletal structure and compaction stability under the same compaction effort.

## 5. Conclusions

This study utilized SmartRock sensors to monitor the acceleration responses of aggregates and compaction degree variations in the top, middle, and bottom surface courses of asphalt pavement during compaction. The aim was to find and establish the correlation between aggregate acceleration characteristics and compaction characteristics of asphalt mixtures from a particle perspective. Based on field experiments and analysis, the following conclusions can be drawn:

- Vertical compression and horizontal shear are the main causes of densification in asphalt mixtures, with vertical compression playing a dominant role. The rolling of steel drums induces a large change in the aggregate acceleration along the rolling direction of rollers, and horizontal shear mainly occurs in the rolling direction.
- The compaction process can be divided into three phases: initial compaction, mid-term compaction, and compaction stabilization. Under the compacted conditions and materials of the paper, the optimal number of vibratory rolling passes for the top, middle, and bottom surface courses are the fourth, sixth, and sixth pass, respectively.
- The vibration waves gradually attenuate on both sides of the vibrating drum as the propagation distance increases, showing a trend of normal distribution. The thickness and gradation of the asphalt surface course have a significant impact on the vibration acceleration, and decreasing the thickness and gradation will result in a decrease in the aggregate acceleration.
- The acceleration indicators representing the compactness of the middle, bottom surface courses lag behind the compaction degree indicators, and the changes in aggregate acceleration can serve as indicators for evaluating the variation of the skeleton structure and compaction conditions.
- There is a linear correlation between the triaxial acceleration of aggregates and compaction degree of asphalt mixtures. The vertical direction of aggregate acceleration has the strongest correlation with compaction degree, and it is recommended to use the linear correlation formula (compaction degree = *a* − *b* × vertical acceleration amplitude, where *a* and *b* are fitting parameters) between vertical acceleration and compaction degree to predict the compaction degree. Furthermore, the slope of the fitting curve can serve as an indicator of the difficulty in forming the compacted skeleton structure and compaction stability of the mixture.

In comparison with the findings of other researchers, our study provides valuable insights into the relationship between aggregate acceleration characteristics and compaction characteristics of asphalt mixtures. The strengths of our methodology lie in the utilization of SmartRock sensors to monitor acceleration responses during compaction, allowing for a more accurate and detailed analysis of the compaction process. However, it is important to acknowledge the limitations of this study, such as the specific conditions and materials used, which may affect the generalizability of the results.

Regarding the future goals of this work, the research topic of this paper will continue to be studied in order to further explore and refine the correlation between aggregate acceleration characteristics and compaction characteristics of asphalt mixtures. Furthermore, further research and investigation will continue exploring the relationship between asphalt mixture properties (such as density, modulus, stiffness, etc.) and particle motion (acceleration frequency, acceleration amplitude, and rotation angle). This will help further understand the complex behavior of asphalt mixtures during compaction. Additionally, the ultimate research goal is to develop models and algorithms based on these findings to predict pavement compaction quality, paving the way for achieving true intelligent compaction (IC).

## Figures and Tables

**Table 1:** The basic technical index of asphalt mixtures.

Mixture Type	Optimal Asphalt Content (%)	VMA (%)	VFA (%)
AC-13C	4.9	14.6	69.5
AC-20C	4.3	12.3	67.6
AC-25C	3.8	12.2	65.4

## Data Availability

The data presented in this study are available upon request from the corresponding author.
